# Double the Prevalence of Stage 2 Hypertension Readings in a Small Group of American Pre-clinical Medical Students Compared to Young Adults Diagnosed with Stage 2 Hypertension in the United States

**DOI:** 10.7759/cureus.7448

**Published:** 2020-03-28

**Authors:** Daniel Mok, Jacek Bednarz, Jan Zieren, Theresa Ferguson, Jordan Glass, Kelcie Smith, Brian Yonish

**Affiliations:** 1 Family Medicine, Lincoln Memorial University - DeBusk College of Osteopathic Medicine, Harrogate, USA

**Keywords:** hypertension, medical, students, american, sleep, waist circumference

## Abstract

Objective

The aim of this study is to compare the prevalence of hypertension (HTN) in pre-clinical (first- and second-year) medical students at Lincoln Memorial University to that of the United States population and identify risk factors in this group.

Materials and Methods

Students from the DeBusk College of Osteopathic Medicine completed a survey that queried age, gender, tobacco use, alcohol consumption, diet, aerobic exercise, mental health, social support, amount of sleep per night, and past medical history. Omron BP710N (Omron 3 series) sphygmomanometers were used to measure blood pressures in the left arm. Waist circumference was measured around the umbilicus. HTN stages were defined under the 2017 ACC/AHA guidelines. Univariate, binominal, and multinomial regression analyses of risk factors were performed using SPSS v22.0 with α = 0.05.

Results

Of the 213 students surveyed, 49.8% (106/213) were males, 49.3% (105/213) were females, and 0.9% (2/213) declined to reveal their gender. The mean age of the sample was 25.8 years (SD = 2.75 years) and the range was between 21 and 37 years. Under the 2017 ACC/AHA guidelines, 36.6% (78/213) were normotensive; 16.4% (35/213) had elevated blood pressure; 29.1% (62/213) had stage 1 HTN; and 17.8% (38/213) had stage 2 HTN. A multinomial logistic regression model was significant, *χ*^2^(9) = 82.934, *p* < 0.001, explained 34.9% (Nagelkerke R^2^) of the variance in HTN, and correctly classified 50.2% of cases. In comparison to normotensive females, normotensive males are 2.81 times more likely (95% CI: 1.04-7.61; *p* = 0.042) to develop stage 2 HTN; increasing waist circumference by 1 cm in normotensive students was associated with a 10% increase (95% CI: 1.06-1.15; *p *< 0.001) in developing stage 2 HTN; and sleeping <6 hours per night was associated with 4.33 times increased (95% CI: 1.52-12.34; *p* = 0.006) likelihood of developing stage 2 HTN with respect to normotensive students who sleep for 6-8 hours a night.

Conclusion

Our sample of medical students has a 2.4 times higher prevalence of stage 2 HTN readings in comparison to adults aged 18-39 according to the 2015-2016 national CDC hypertension prevalence report. Risk factors including male gender and sleeping less than 6 hours per night are significant predictors of elevated and stage 2 HTN. Waist circumference is predictive of stage 1 HTN and stage 2 HTN. Additional studies should be conducted to increase the sample size in order to better assess the prevalence of stage 2 HTN in American medical students.

## Introduction

In a 2015-2016 study, The Centers of Disease Control and Prevention (CDC) estimates that 7.5% of the US adults of age 18-39 years have high blood pressure, which they defined as a systolic pressure greater than or equal to 140 mm Hg or diastolic pressure greater than or equal to 90 mm Hg [1]. As of today, the aforementioned blood pressure criteria meet the definition for stage 2 HTN under the relatively new 2017 American College of Cardiology (ACC)/American Heart Association (AHA) guidelines. These are the first changes to hypertension guidelines since 2003, which have lowered the threshold in defining hypertension. Hypertension now encompasses elevated, stage 1 HTN, stage 2 HTN, and hypertensive crisis. Individuals who met the stage 1 HTN criteria before 2017 are now classified as having stage 2 HTN based on the 2017 guidelines. In addition, the category of prehypertension has been eliminated and replaced with either elevated or stage 1 HTN. Normal is defined as a systolic pressure less than 120 mm Hg and diastolic pressure less than 80 mm Hg; elevated is a systolic pressure of 120-129 mm Hg and diastolic pressure less than 80 mm Hg; stage 1 HTN is a systolic pressure of 130-139 mm Hg and/or diastolic pressure of 80-89 mm Hg; stage 2 HTN is a systolic pressure of 140 mm Hg and greater and/or a diastolic pressure of 90 mm Hg and greater; a hypertensive crisis is defined as a systolic pressure greater than 180 mm Hg and/or a diastolic pressure greater than 120 mm Hg. Due to these changes in the guidelines, it is now estimated that 46% of the US population has hypertension [2]. 

Research on HTN involving American medical students is lacking. This is one of the earliest known studies to survey blood pressures of American medical students and classify the readings under the 2017 ACC/AHA guidelines in an attempt to understand the prevalence of HTN in this population. It is important to understand this population’s blood pressure, as first- and second-year medical students are at risk to acquire modifiable risk factors due to the nature of the education. Students are sedentary for most of the day, experience significant amounts of stress, and lack time to exercise and to cook healthy meals. There have been studies that looked at the blood pressure in the African, Middle Eastern, and South-East Asian medical students [3-5]. These studies have found high blood pressure among students; however, reported prevalence varies wildly among studies. 

It is well known that many individuals are unaware of having HTN due to the lack of symptoms from the disorder, and that HTN in young adults is a predisposition for developing cardiovascular disease [6]. Early identification is crucial because a physician can help a patient modify behaviors in order to stop the progression of the disorder. Hypertension independently increases the risk of cardiovascular disease even after adjusting for multiple cardiovascular risk factors such as total cholesterol, smoking status, body mass index (BMI), and diabetes mellitus [6]. Therefore, because HTN goes unnoticed and HTN independently elevates risk for cardiovascular disease, it is important for young adults with modifiable risk factors to monitor their blood pressure and make changes to their behavior. 

It seems that HTN may be a problem in medical students; therefore, this study’s goal will be to compare the prevalence of HTN readings in pre-clinical (first and second year) medical students at the Lincoln Memorial University to that of the US population and identify risk factors in this group. 

## Materials and methods

Design and ethical considerations 

First- and second-year medical students were surveyed during the 2018-2019 academic year. The population of the first- and second-year students enrolled at the Lincoln Memorial University was 460 students. In order to achieve a confidence level of 95% with a 5% margin of error and a 50% population proportion for this population, a target sample size of 210 students was necessary according to Cochran’s sample size formula. 

The Institutional Review Board at Lincoln Memorial University approved this study, protocol number: 756 V.0. The aim of this study and protocol to obtain physical measurements were explained to students. Written consent was obtained from each participant. Students were reassured that participation was in no way mandatory and students had the right to withdraw from the study at any time with no consequences. 

Setting

The study was conducted in Harrogate, Tennessee at Lincoln Memorial University - DeBusk College of Osteopathic Medicine. 

Sample

First- and second-year medical students were recruited to participate after lectures, tutorials, and team-based learning exercises. Eligibility criteria included being a first- or second-year medical student over the age of 18. Exclusion criteria included tobacco use 30 minutes prior to blood pressure measurement and any physical exercise 30 minutes prior to blood pressure measurement. Caffeine intake was not considered an exclusionary criterion for this study. Regular caffeine intake does increase blood pressure in the long term [7]. However, individuals who chronically drink caffeinated beverages do not experience acute spikes in blood pressure after ingestion [8].

Data collection

**Figure 1:**
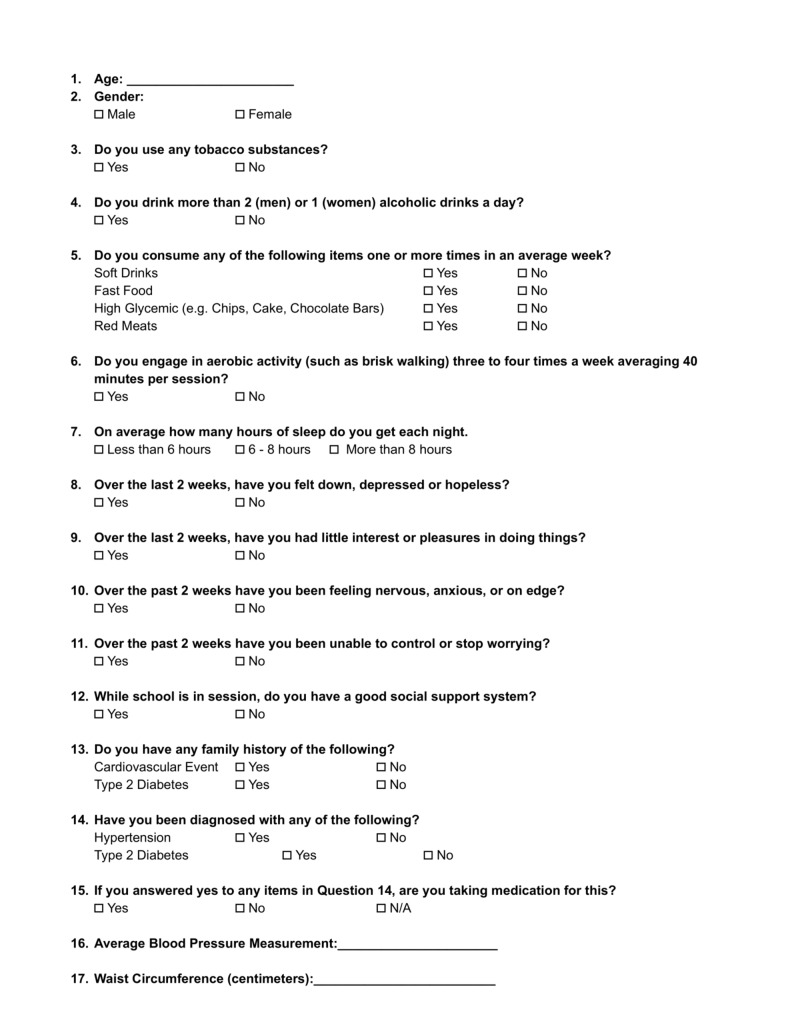
Blank sample questionnaire

Participants were asked to fill out a paper questionnaire prior to having their blood pressure and waist circumference measured (Figure 1). The questionnaire queried age; gender; tobacco use; alcohol use; consumption of soft drinks, fast food, high glycemic foods, and red meats; aerobic exercise; mental health; social support; amount of sleep per night; history of diabetes; history of hypertension; and family history. To identify individuals at risk for major depressive disorder (MDD), we asked participants if they have felt down, depressed, or hopeless and if they have little interest or pleasure in doing things over the last two weeks. Answering yes to both these two questions has a sensitivity of 83% and specificity of 92% for detecting MDD [9]. To identify individuals at risk for generalized anxiety disorder (GAD), we asked participants if they have been feeling nervous anxious or on edge and if they have been unable to control or stop worrying for the past 2 weeks [10]. Answering yes to both the two previous questions would deem the participant at risk for GAD.

Blood pressure was measured by six trained researchers using Omron BP710N sphygmomanometers. Researchers practiced taking blood pressures prior to surveying students in order to ensure measurement accuracy. In order to minimize the effects of white coat hypertension, we used automated blood pressure cuffs to reduce clinician-participant anxiety and we took multiple readings separated in time. Blood pressures were measured according to the AHA’s proper seated office blood pressure measurement guidelines with some modifications [11]. Participants were asked to relax and sit in a chair with their feet flat on the floor and back supported. Prior to measuring a participant’s blood pressure, a paper questionnaire was given to the participant to fill out. If participants had additional queries with respect to any question on the questionnaire, participants were told; that no additional information could be provided and that they could either answer as they had interpreted the question or skip the question altogether. Blood pressure readings were taken when the participant had been seated for equal to or more than 3 minutes. Researchers were instructed that neither they nor the participant was to talk while the machine was taking a measurement. Participants’ left arms were supported via a table that was at the height of their heart during measurement. After the first measurement was conducted, participants were asked to rest for 1-2 minutes before a second blood pressure reading was taken. A third measurement was taken 1-2 minutes after the second blood pressure reading if the difference in systolic or diastolic pressure between the first and second readings exceeded 10 mm Hg. The average of the two or three blood measurements was recorded for the individual.

A majority of the procedural methodologies in this study emulated that of the 2015-2016 CDC study to ensure that the data of this study would be comparable to that of the CDC [1]. Measuring blood pressure at only one point in time, inclusion/exclusion criteria, and measurement protocol were replicated. It was noted in the CDC’s procedural methodology that measurements were obtained during a single examination visit. In this study, 2-3 blood pressure measurements were also taken during one encounter. In the CDC’s determination of HTN prevalence, the exclusion criteria included those younger than 18 years of age, pregnant, had rashes, gauze dressings, casts, edema, paralysis, tubes, open sores or wounds, withered arms, a-v shunts, or radical mastectomy. 

Waist circumference was measured with a tape measure wrapped above the iliac crests around the level of the umbilicus. Measurements were done directly on the skin; however, if the student requested, a measurement was taken over a T-shirt. Participants were asked to inhale and exhale after the tape measure was wrapped snug around the waist. Measurement was taken after the participant finished exhaling [12]. Measurements were recorded to the nearest centimeter. 

Statistical analysis 

The data were analyzed using SPSS v22.0 to tally the frequency of the risk factors and blood pressure categories. A binomial logistic regression analysis was used to test significant risk factors for hypertension. Hypertension and normotension were the nominal variables and the independent risk factors were waist circumference, age, gender, tobacco use, alcohol use, soft drink consumption, fast food consumption, high glycemic food consumption, red meat consumption, aerobic exercise, feeling depressed, anhedonia, anxiety, uncontrolled worrying, major depressive disorder, generalized anxiety disorder, and social support. A multinomial logistic regression was also used where the nominal variables were normotension, elevated, stage 1 HTN, and stage 2 HTN and the independent risk factors were the significant risk factors obtained from the binomial regression analysis. A Chi-square test was used to assess the difference in the proportions of risk factors in corresponding blood pressure categories and genders. Independent t-tests were used to compare the mean waist circumference, systolic pressure, and diastolic pressure with respect to gender. 

## Results

Description of sample

The sample was made up of 213 students of which 49.8% (106/213) were males, 49.3% (105/213) were females, and 0.9% (2/213) declined to reveal their gender. The mean age of the sample was 25.8 years (SD = 2.75 years) and the range was 21-37 years. Under the 2017 ACC/AHA guidelines 36.6% (78/213) were normotensive; 16.4% (35/213) had elevated blood pressure; 29.1% (62/213) had stage 1 HTN; and 17.8% (38/213) had stage 2 HTN. Only 2.3% (5/212) of students have a known diagnosis of hypertension and 0.5% (1/212) have a known diagnosis of type 2 diabetes mellitus. There were 11 surveys out of the 213 which had at least one unanswered question. Therefore, 202 complete surveys were used in the binomial and multinomial regression analysis. In univariate analysis, all answered questions from the 213 surveys were used. 

ACC/AHA hypertension levels & associated risk factors

Univariate analysis of modifiable and nonmodifiable risk factors in normotensive, elevated, stage 1 HTN and stage 2 HTN categories showed statistically significant differences in waist circumference (*X*^2^(12, *N* = 213) = 52.227, *p* < 0.001); gender (*X*^2^(3, *N* = 211) = 30.091, *p* < 0.001); tobacco use (*X*^2^(3, *N* = 211) = 8.798, *p* = 0.032); fast food consumption (*X*^2^(3, *N* = 210) = 7.882, *p* = 0.049); and amount of sleep per night (*X*^2^(6, *N* = 212) = 19.355, *p* = 0.004). (Table 1)

**Table 1:** Distribution of each risk factor in its corresponding blood pressure category Blood pressure readings (n = 213) were taken from students where 11 students did not answer at least one survey question. Pearson's chi-square was performed for each risk factor. *p < 0.05

	Normotensive		Elevated		Stage 1		Stage 2		Test Statistic	P	Total	
	Count	%	Count	%	Count	%	Count	%			Count	%
Waist Circumference (cm)												
60-69	16	20.5	2	5.7	5	8.1	2	5.3	52.227*	<0.001*	25	11.7
70-79	30	38.5	12	34.3	13	21	1	2.6			56	26.3
80-89	18	23.1	16	45.7	19	30.6	10	26.3			63	29.6
90-99	11	14.1	4	11.4	16	25.8	14	36.8			45	21.1
≥100	3	3.8	1	2.9	9	14.5	11	28.9			24	11.3
Age												
21-23	15	19.2	5	14.3	12	19.4	6	15.8	13.654	0.552	38	17.8
24-26	41	52.6	21	60	31	50	17	44.7			110	51.6
27-29	17	21.8	7	20	13	21	9	23.7			46	21.6
30-31	2	2.6	1	2.9	3	4.8	3	7.9			9	4.2
32-33	1	1.3	1	2.9	3	4.8	0	0			5	2.3
>33	2	2.6	0	0	0	0	3	7.9			5	2.3
Gender												
Male	21	27.3	26	74.3	34	55.7	26	68.4	30.091*	<0.001*	107	50.7
Female	56	72.7	9	25.7	27	44.3	12	31.6			104	49.3
Tobacco Use												
Yes	1	1.3	1	2.9	7	11.5	1	2.6	8.798*	0.032*	10	4.7
No	76	98.7	34	97.1	54	88.5	37	97.4			201	95.3
Alcohol Use												
Yes	0	0	1	2.9	4	6.7	2	5.3	5.246	0.155	7	3.3
No	78	100	34	97.1	56	93.3	36	94.7			204	96.7
Soft Drinks												
Yes	27	34.6	13	37.1	30	49.2	18	48.6	4.048	0.256	88	41.7
No	51	65.4	22	62.9	31	50.8	19	51.4			123	58.3
Fast Food												
Yes	33	42.3	18	51.4	30	50	26	70.3	7.882*	0.049*	107	51
No	45	57.7	17	48.6	30	50	11	29.7			103	49
High Glycemic												
Yes	50	64.1	22	62.9	34	56.7	29	76.3	3.921	0.27	135	64
No	28	35.9	13	37.1	26	43.3	9	23.7			76	36
Red Meats												
Yes	35	44.9	23	65.7	35	58.3	24	63.2	6.142	0.105	117	55.5
No	43	55.1	12	34.3	25	41.7	14	36.8			94	44.5
Aerobic Exercise												
Yes	36	46.8	18	52.9	32	52.5	17	44.7	0.935	0.817	103	49
No	41	53.2	16	47.1	29	47.5	21	55.3			107	51
Amount of Sleep												
<6	11	14.1	13	37.1	15	24.6	15	39.5	19.355*	0.004*	54	25.5
6 to 8	60	76.9	20	57.1	46	75.4	23	60.5			149	70.3
>8	7	9	2	5.7	0	0	0	0			9	4.2
Major Depressive Disorder												
Yes	10	12.8	4	11.4	10	16.4	9	23.7	2.84	0.417	33	15.6
No	68	87.2	31	88.6	51	83.6	29	76.3			179	84.4
Feeling Depressed												
Yes	19	24.4	7	20	14	23	11	28.9	0.858	0.836	51	24.1
No	59	75.6	28	80	47	77	27	71.1			161	75.9
Loss of Pleasure												
Yes	14	17.9	5	14.3	10	16.4	12	31.6	4.658	0.199	41	19.3
No	64	82.1	30	85.7	51	83.6	26	68.4			171	80.7
Generalized Anxiety Disorder												
Yes	16	20.5	8	22.9	15	24.6	6	15.8	1.164	0.762	45	21.2
No	62	79.5	27	77.1	46	75.4	32	84.2			167	78.8
Uncontrolled Anxiety												
Yes	50	64.1	26	74.3	38	62.3	21	55.3	2.923	0.404	135	63.7
No	28	35.9	9	25.7	23	37.7	17	44.7			77	36.3
Worrying												
Yes	18	23.1	8	22.9	16	26.2	6	15.8	1.476	0.688	48	22.6
No	60	76.9	27	77.1	45	73.8	32	84.2			164	77.4
Social Support												
Yes	73	93.6	33	94.3	57	93.4	35	92.1	0.153	0.985	198	93.4
No	5	6.4	2	5.7	4	6.6	3	7.9			14	6.6
Family History: Cardiovascular Event												
Yes	41	52.6	17	48.6	27	45	19	50	0.791	0.852	104	49.3
No	37	47.4	18	51.4	33	55	19	50			107	50.7
Family History: Type 2 Diabetes												
Yes	32	41	17	48.6	23	38.3	16	42.1	0.977	0.807	88	41.7
No	46	59	18	51.4	37	61.7	22	57.9			123	58.3

In the binomial logistic regression model where the dichotomous outcome variable was hypertension in reference to normotension, the significant predictive risk factors were found to be waist circumference (OR: 1.04; 95% CI: 1.004-1.081; *p* = 0.028); male gender (OR: 1.351; 95% CI: 1.662-8.963; *p* = 0.002); and sleeping under 6 hours (OR: 3.547; 95% CI: 1.511-8.328; *p* = 0.004; Table 2).

**Table 2:** Binomial regression of the risk factors of hypertension (encompassing elevated, stage 1 HTN, and stage 2 HTN) compared to normotension Omnibus chi-square = 61.572, *P *< 0.001; Nagelkerke *R*^2^: 0.359. The model predicts 74.8% of cases correctly (*n *= 202). *B* is the coefficient for the respective risk factor in the regression. *p < 0.05 † SPSS could not calculate an upper CI interval. CI, confidence interval

Risk Factors	B	Significance	Odds Ratio	95% Confidence Interval	
				Lower	Upper
Waist Circumference	0.041	0.028*	1.04	1.004	1.081
Age	-0.061	0.376	0.94	0.823	1.077
Male Gender	1.351	0.002*	3.86	1.662	8.963
Tobacco Use	1.251	0.333	3.5	0.278	43.969
Alcohol Use	21.626	0.999	2.5x10^2	0	†
Soft Drinks	0.443	0.269	1.56	0.71	3.414
Fast Food	-0.074	0.854	0.93	0.423	2.04
High Glycemic	-0.026	0.949	0.98	0.445	2.136
Red Meats	0.441	0.264	1.56	0.717	3.369
Aerobic Exercise	0.001	0.997	1	0.461	2.176
<6 hours of sleep	1.266	0.004*	3.55	1.511	8.328
Major Depressive Disorder	1.487	0.203	4.43	0.448	43.748
Feeling Depressed	-0.425	0.502	0.65	0.19	2.257
Loss of Pleasure	-1.223	0.181	0.29	0.049	1.766
Generalized Anxiety Disorder	1.268	0.442	3.56	0.14	90.167
Uncontrolled Anxiety	-0.103	0.806	0.9	0.397	2.049
Worrying	-0.801	0.605	0.45	0.022	9.312
Social Support	-0.407	0.607	0.67	0.141	3.14
Family History of Cardiovascular Event	-0.261	0.498	0.77	0.362	1.638
Family History of Type 2 Diabetes	0.253	0.554	1.29	0.556	2.982

Further multinomial logistic regression analysis of the significant predictors from the binomial regression showed that male gender is a significant predictor of elevated (OR: 11.50; 95% CI: 3.92-33.80; p < 0.001) and stage 2 HTN (OR: 2.81; 95% CI: 1.04-7.61; *p* = 0.042). Sleeping less than 6 hours per night is also a significant predictor of elevated (OR: 4.96; 95% CI: 1.77-13.92; *p* = 0.002) and stage 2 HTN (OR: 4.33; 95% CI: 1.52-12.34; *p* = 0.006). Increasing waist circumference by 1 cm is predictive of stage 1 HTN (OR: 1.05; 95% CI: 1.02-1.09; *p* = 0.006) and stage 2 HTN (OR: 1.10; 95% CI: 1.06-1.15; *p* < 0.001) for this sample (Table 3).

**Table 3:** Multinomial regression of predictive risk factors of elevated, stage 1 HTN, and stage 2 HTN compared to normotension. Likelihood ratio chi-square test = 82.934; *p *< 0.001; Nagelkerke *R*^2^: 0.349. The model predicts 50.2% of cases correctly (*n* = 202). *B* is the coefficient for the respective risk factor in the regression. *p < 0.05.

	B	Significance	Odds Ratio	95% Confidence Interval	
				Lower	Upper
Elevated Blood Pressure					
Waist Circumference	-0.023	0.371	0.98	0.93	1.03
Male Gender	2.443	<0.001^*^	11.5	3.92	33.8
<6 hours of sleep	1.602	0.002^*^	4.96	1.77	13.92
Stage 1 Hypertension					
Waist Circumference	0.052	0.006^*^	1.05	1.02	1.09
Male Gender	0.8	0.051	2.23	1	4.96
<6 hours of sleep	0.786	0.092	2.19	0.88	5.48
Stage 2 Hypertension					
Waist Circumference	0.098	<0.001^*^	1.1	1.06	1.15
Male Gender	1.034	0.042^*^	2.81	1.04	7.61
<6 hours of sleep	1.466	0.006^*^	4.33	1.52	12.34

Differences in gender

In this study, males made up a larger proportion of students with elevated, stage 1 HTN and stage 2 HTN, while females were the larger portion of normotensive students (Table 1). The mean abdominal circumference for the 106 males (M = 89.72 cm, SD = 11.77) compared to the 105 females (M = 78.89 cm, SD = 11.14) was significantly larger, t(209) = 6.860, *p* < 0.001. The systolic pressure for the 106 males (M = 129.11 mmHg, SD = 12.43) compared to the 105 females (M = 115.95, SD = 11.73) was significantly larger, t(209) = 7.909, *p* < 0.001. However, the diastolic pressure for the 106 males (M = 80.04 mmHg, SD = 8.66) compared to the 105 females (M = 78.51, SD = 9.16) showed no statistically significant difference, t(209) = 1.242, *p* = 0.216. Univariate analysis showed statistically significant differences in the blood pressure (*X*^2^(3, *N *= 211) = 28.295, *p* < 0.001); waist circumference (*X*^2^(4, *N* = 211) = 47.509, *p* < 0.001); soft drink consumption (*X*^2^(1, *N* = 210) = 5.007, *p* = 0.025); fast food consumption (*X*^2^(1, *N* = 209) = 7.291, *p* = 0.007); aerobic exercise engagement (*X*^2^(1, *N* = 209) = 5.216, *p* = 0.022); generalized anxiety disorder (*X*^2^(1, *N* = 211) = 6.538, *p* = 0.011); worrying (X^2^(1, *N* = 211) = 7.102, *p* = 0.008); family history of a cardiovascular event (*X*^2^(1, *N* = 210) = 6.172, *p* = 0.013); and family history of type 2 diabetes (X^2^(1, *N* = 210) = 5.007, *p* = 0.025) between males and females (Table 4).

**Table 4:** Distribution of blood pressure categories and risk factors in males and females A total of 106 males and 105 females were surveyed (*n* = 211), where 9 students did not answer at least one survey question. Pearson Chi-square was performed for each risk factor. *p < 0.05

	Male		Female		Test Statistic	P	Male and Female Combined	
	Count	%	Count	%			Count	%
Blood Pressure								
Normotensive	21	19.8	56	53.3	28.295*	<0.001*	77	36.5
Elevated	25	23.6	10	9.5			35	16.6
Stage 1 Hypertension	34	32.1	27	25.7			61	28.9
Stage 2 Hypertension	26	24.5	12	11.4			38	18
Waist Circumfernce (cm)								
60-69	3	2.8	22	21	47.509*	<0.001*	25	11.8
70-79	14	13.2	42	40			56	26.5
80-89	41	38.7	21	20			62	29.4
90-99	29	27.4	15	14.3			44	20.9
≥100	19	17.9	5	4.8			24	11.4
Age								
21-23	12	11.3	26	24.8	8.583	0.127	38	18
24-26	57	53.8	52	49.5			109	51.7
27-29	24	22.6	21	20			45	21.3
30-31	6	5.7	3	2.9			9	4.3
32-33	3	2.8	2	1.9			5	2.4
>33	4	3.8	1	1			5	2.4
Tobacco Use								
Yes	7	6.7	3	2.9	1.68	0.195	10	4.8
No	98	93.3	102	97.1			200	95.2
Alcohol Use								
Yes	2	1.9	5	4.8	1.33	0.249	7	3.3
No	103	98.1	100	95.2			203	96.7
Soft Drinks								
Yes	52	49.5	36	34.3	5.007*	0.025*	88	41.9
No	53	50.5	69	65.7			122	58.1
Fast Food								
Yes	63	60.6	44	41.9	7.291*	0.007*	107	51.2
No	41	39.4	61	58.1			102	48.8
High Glycemic								
Yes	67	63.8	67	63.8	0	1	134	63.8
No	38	36.2	38	36.2			76	36.2
Red Meats								
Yes	74	70.5	43	41	18.547*	<0.001*	117	55.7
No	31	29.5	62	59			93	44.3
Aerobic Exercise								
Yes	60	57.1	43	41.3	5.216*	0.022*	103	49.3
No	45	42.9	61	58.7			106	50.7
Amount of Sleep								
<6	25	23.6	29	27.6	0.511	0.775	54	25.6
6 to 8	76	71.7	72	68.6			148	70.1
>8	5	4.7	4	3.8			9	4.3
Major Depressive Disorder								
Yes	17	16	16	15.2	0.26	0.873	33	15.6
No	89	84	89	84.8			178	84.4
Feeling Depressed								
Yes	22	20.8	29	27.6	1.356	0.244	51	24.2
No	84	79.2	76	72.4			160	75.8
Loss of Pleasure								
Yes	23	21.7	18	17.1	0.699	0.403	41	19.4
No	83	78.3	87	82.9			170	80.6
Generalized Anxiety Disorder								
Yes	15	14.2	30	28.6	6.538*	.011*	45	21.3
No	91	85.8	75	71.4			166	78.7
Uncontrolled Anxiety								
Yes	63	59.4	72	68.6	1.911	0.167	135	64
No	43	40.6	33	31.4			76	36
Worrying								
Yes	16	15.1	32	30.5	7.102*	0.008*	48	22.7
No	90	84.9	73	69.5			163	77.3
Social Support								
Yes	97	91.5	100	95.2	1.184	0.277	197	93.4
No	9	8.5	5	4.8			14	6.6
Family History: Cardiovascular Event								
Yes	43	41	61	58.1	6.172*	0.013*	104	49.5
No	62	59	44	41.9			106	50.5
Family History: Type 2 Diabetes								
Yes	36	34.3	52	49.5	5.007*	0.025*	88	41.9
No	69	65.7	53	50.5			122	58.1

## Discussion

We studied the prevalence of 2017 ACC/AHA hypertension categories and associated risk factors in first- and second-year medical students at Lincoln Memorial University - DeBusk College of Osteopathic Medicine. We found that about two-thirds of the sample had hypertensive readings and that 17.8% (38/213) of students had readings that fit the criteria of stage 2 HTN. This is concerning given that the prevalence of stage 2 HTN in adults of 18-39 years of age in the United States is estimated to be about 7.5% by the CDC 2015-2016 report [1]. Hypertension was once thought of as a chronic disease associated with the elderly population; however, it is now apparent that incidence among young adults (18-30 years of age) is becoming an issue [13]. The etiology of the increased prevalence is not completely understood; it is known that certain risk factors and diseases such as decreased physical activity, diabetes, depressive states, and central obesity are becoming more prevalent in young adults [13-15]. Medical students may be a subset of the young adult population at an increased risk of developing hypertension due to risk factors that are known to already affect this population. 

In this study, we found that students who sleep less than six hours per night are at significant risk of developing elevated, stage 1 HTN, and stage 2 HTN. Studies have shown that inadequate sleep and poor sleep quality are associated with increased blood pressure [16-17]. In our study, 25.5% (54/212) of students sleep an average of 6 hours or less per night, and we believe that the issue of sleep deprivation is prevalent in other medical schools. In recent work looking at medical students at SUNY Downstate Medical School, researchers found that 84.7% of students slept < 7 hours a day during the week of an examination [18]. The study concluded that a majority of SUNY medical students are sleeping an inadequate amount during their clinical and pre-clinical years. Another study at a large U.S. public university found that over one-third of students sleep less than 7 hours and about the same proportion pull at least one all-nighter in an academic year [19]. Although the lack of sleep may be contributing to hypertension in our study, we do not believe it is the only cause of the higher prevalence of HTN. The CDC determined that the percent of American adults sleeping less than 6 hours per night ranged from 27.7% to 39.8% [20]. Our study found that 25.5% of medical students are sleeping less than 6 hours per night. If lack of sleep were to play a major role in causing HTN in our sample, we would expect a greater percentage of students sleeping less than 6 hours per night in comparison to that of the CDC’s data. 

This study also found that an increase in waist circumference was significantly predictive of developing stage 1 HTN and stage 2 HTN. It is known that waist circumference is a strong independent predictor of hypertension [21]. Males with waist size ≥102 cm have a 3.04 times greater probability of being hypertensive than males with waist sizes <94 cm; females with waist sizes ≥88 cm have a two-fold increased risk for hypertension than females with waist sizes <80cm [22]. In our sample, about 18.9% (20/106) of males had a waist circumference of 100 cm or greater, and 18.1% (19/105) of females had a waist circumference of 90 cm or greater. This means that about one fifth (39/211) of male and female students have waist circumferences that increase their likelihood of developing hypertension based on the previously discussed study. Lifestyle factors such as diet and exercise are known to be major variables that influence waist circumference. In our sample, 51.0% (107/210) of students eat fast food at least once a week, and only 49% (103/210) of students engage in an aerobic activity (such as brisk walk) three to four times a week averaging 40 minutes a session. The proportion of students in the sample who consume red meats, high glycemic foods, and soft drinks are similar to that of fast food. 

Finally, being male was a significant predictor of elevated and stage 2 HTN. This finding parallels past research using the National Longitudinal Study of Adolescent to Adult that found among the US young adults, women are far less likely to be hypertensive than men (12% vs 27%) [23]. The same study also suggests that hypertension awareness among young men and women is low, but is especially lower in men. In our sample 63.3% (135/213) had hypertensive readings; however, only 2.3% (5/212) have been diagnosed with hypertension. This discrepancy begs the question if medical students are a subpopulation of young adults that are also unaware of their own blood pressure. In our univariate analysis of risk factors in males and females, we found that men had higher waist circumferences. In addition, a greater proportion of men consumed more soft drinks, fast food, and red meats than women. It could be the case that the relatively poor diet in this sample of male medical students is causing the disparity in the prevalence of hypertension between females and males. 

No statistically significant difference was found in the proportion of students positive for generalized anxiety disorder risk, major depressive disorder risk, feelings of depression, and anhedonia in different blood pressure groups (Table 1). Likewise, binomial logistic regression did not show the previous mental health variables to be predictive of hypertension (Table 2). However, it is worth mentioning that 15.6% (33/212) of the sample are at risk for major depressive disorder and 21.2% (45/212) are at risk for generalized anxiety disorder. The prevalence of MDD in American adults seems to decline with age and is estimated to be 13.1% in adults between 18-25 years of age; 7.7% for adults 26-49 years of age; and 4.7% for those 50 years of age and greater according to the CDC [24]. Interestingly, our sample shows the inverse with a prevalence of 10.7% (12/112) in students of 21-25 years of age and 21% (21/100) in students between 26-37 years of age. In terms of GAD risk in our study, there is a prevalence of 22.3% (25/112) in students of 21-25 years of age, and 20% (20/100) in students of 26-37 years of age. According to the national comorbidity survey, an estimated 2.7% of American adults experience GAD at some point during a calendar year [25]. 

Literature suggests that as a student progresses through medical school, mental health declines [26]. Prevalence estimates of major depressive disorder and feelings of depression in medical students vary largely in studies, but a recent meta-analysis from 167 cross-sectional studies estimates that the prevalence of MDD and feelings of depression is 27.2% in medical students [27]. In our study, there was a significant difference between the proportion of males and females at risk for GAD. In our sample, 28.6% (30/105) of women were at risk for GAD, while only 14.2% (15/106) of men were at risk (X^2^(1, N = 211) = 6.538, p = 0.011). There was no significant difference in MDD between males and females. In terms of MDD, 15.2% (16/105) of women were at risk, while 16.0% (17/106) of men were at risk (X^2^(1, N = 211) = 0.026, p = 0.873). Previous literature has found that there is a positive association between chronic poor mental health and hypertension [28]. However, our study showed no relationship between mental health and hypertension. This discrepancy could be the result of not evaluating for chronic mental health in our sample. 

Even though this study found hypertension in about two-thirds of medical students, limitations need to be considered. Although we were able to take repeated measurements of an individual’s blood pressure, this is not adequate to diagnose someone with hypertension. A diagnosis should only be made after the average of two or more readings are obtained on two or more occasions [2]. In addition, our sample size is small when compared to the estimated 60,000 pre-clinical medical students in the United States [29-30]. Further, the study only surveyed students at one American medical school. Therefore, for these three reasons, the results of this study cannot be generalized. Further studies across various medical schools are currently being implemented to further evaluate the prevalence of HTN. In addition, this is a cross-sectional study, so we did not assess the duration that our chosen risk factors have been present in the sample. The assessment of MDD and GAD was only surveyed in the last two weeks prior to a participant completing a questionnaire. Evaluation of a past history of MDD and GAD may have shown that individuals in our sample with chronic mental health issues have statistically higher rates of hypertension. Finally, all the information obtained from medical students other than waist circumference and blood pressure was self-reported, therefore behaviors may have been over or underestimated.

## Conclusions

A small group of pre-clinical medical students has more than a two times increase in prevalence of stage 2 HTN readings in comparison to Americans 18-39 years diagnosed with stage 2 HTN. The significant predictive risk factors found in this study were sleep, waist size, and gender. The influence these factor have on the wider medical student population is not fully understood. The degree to which these findings may be seen in other medical schools is the larger question postulated upon closing this study. We encourage future work at different medical schools in the United States so there can be a better understanding of the prevalence and risk factors of the of 2017 ACC/AHA hypertension categories in pre-clinical medical students. 
